# Molecular and Microbial Detections of *Streptococcus mutans* and Lactobacilli in Dental Caries: An Experimental Study on Iranian Children Aged 5–9

**DOI:** 10.1002/cre2.70039

**Published:** 2024-12-12

**Authors:** Marzieh Danaei, Milad Mollaali, Vida Fakharmohialdini, Hamidreza Poureslami, Fatemeh Sadat Sajadi, Elham Farokh Gisour, Fatemeh Jahanimoghadam, Aida Gholampour, Mehrnaz Foroudisefat, Arezoo Mirshekari, Raziyeh Shojaeipour

**Affiliations:** ^1^ Dana Gene Pajoohan Karmania Company, Member of Iran High‐Tech Laboratory Network Kerman Iran; ^2^ Department of Biology, Faculty of Science University of Sistan and Baluchestan Zahedan Iran; ^3^ Oral and Dental Diseases Research Center, Department of Pediatrics Dentistry, School of Dentistry Kerman University of Medical Sciences Kerman Iran; ^4^ Social Determinants on Oral Health Research Center Kerman University of Medical Sciences Kerman Iran; ^5^ Department of Pediatrics Dentistry, School of Dentistry Kerman University of Medical Sciences Kerman Iran

**Keywords:** Bacterial load, Dental care for children, Dental caries, Dental caries activity tests

## Abstract

**Objectives:**

Lactobacilli and *Streptococcus mutans* are stigmatized as cariogenic bacteria, but few studies have simultaneously examined the quantitative and qualitative aspects of lactobacilli and *S. mutans* in childhood dental caries. Therefore, this study aimed to detect the presence of *S. mutans* and lactobacilli in dental caries using Snyder's test, colony counting, and PCR in the primary teeth of Iranian children with dental caries.

**Material and Methods:**

A cross‐sectional study was conducted in Kerman, Iran, from March to Jun 2024. After dental examinations, 120 eligible children aged 5–9 were chosen using simple random sampling and classified into four groups based on their decayed, missing, and filled (primary) teeth (dmft) index: Group 1 (dmft = 0), Group 2 (dmft = 4–6), Group 3 (dmft = 7–9), and Group 4 (dmft = 10–13). The salivary levels of lactobacilli and *S. mutans* were calculated using colony counting (CFU/ml). Moreover, Snyder's test was applied to evaluate caries activity. PCR was also performed for molecular detection of lactobacilli (*16S rRNA* gene) and *S. mutans (gftB* gene). Lastly, the association between bacterial counting, molecular findings, and Snyder's test was estimated through statistical methods using SPSS 27.

**Results:**

Significant differences were found between the age and the PCR results of lactobacilli and *S. mutans* among all dmft Groups (*p* < 0.05). Moreover, positive significant correlations were observed between the counts of lactobacilli and *S. mutans* in dmft Group 1 compared to other dmft Groups (*p* < 0.05). Furthermore, the counts of these two bacteria differed significantly in Snyder's test (*p* < 0.05). However, Snyder's test differed significantly from the *S. mutans* PCR (*p* < 0.05), but not from the lactobacilli PCR (*p* > 0.05).

**Conclusions:**

The results of the study could potentially be considered a promising and cost‐effective screening program to identify children who are susceptible to dental caries.

## 1 Introduction

Dental caries represents a preventable non‐communicable disease that affects a significant portion of the population across their lifespan (Pitts et al. 2021). Caries prevention has traditionally depended on fluoride exposure, diet control, oral hygiene status, and consuming antibacterial agents. In 2019, dental caries was estimated to be 43% prevalent worldwide and 46% prevalent in low‐income countries (Spatafora et al. 2024).

Since oral and dental hygiene is directly influenced by the change in the natural microbial flora and its identification is faster and more accessible in the early stages of dental caries, it is considered an ideal option for screening children exposed to dental caries (Sedghi et al. 2021). Previous studies have often addressed qualitative or quantitative identification of microbes involved in dental caries, and very few research works, especially in Iran, have addressed both qualitative and quantitative aspects of important bacteria in dental caries. To date, no study has been conducted to identify *Streptococcus mutans* and lactobacilli bacteria in children in Kerman Province; children's demographic and lifestyle factors associated with dental caries are also not fully discussed. Globally, Snyder's test has been examined as a complementary test in some studies along with the evaluation of *S. mutans*. Therefore, providing rapid and inexpensive dental caries testing to detect at‐risk children in low‐ and middle‐income countries could be a reliable strategy (Nguyen et al. 2023).

A systematic review study recently reported that primary dental caries rates declined in children aged 2–5 years in the United States from 1988 to 2016; however, no obvious declining tendencies were seen in low‐ and middle‐income nations because of a rise in sugar intake (Spatafora et al. 2024).

The global prevalence of early childhood caries is estimated at 49%. The prevalence varies in different countries; for example, in Turkey, Iraq, and Qatar, it is 73.8%, 77.8%, and 89.2%, respectively. However, in Greece and Japan, it is 19.3% and 20.6%, respectively (Maklennan et al. 2024). In Iran, the prevalence is reported to be 72.8% (Soltani et al. 2020). A study in Iran stated that the decayed, missing, and filled teeth (dmft) index has approximately 17% increased in Iranian children aged 5–9 years from 1990 to 2017 (Shoaee et al. 2022). Also, dmft/DMFT score has increased among the Iranian population by more than 15% from 1990 to 2017; particularly, this dmft/DMFT index in Kerman Province has increased considerably from approximately 4.53 in 1990 to over 5.24 in 2017 (Shoaee et al. 2024; Shoaee et al. 2022). Additionally, it has been reported that high dental expenditures are the primary obstacle to children's access to oral health care in Kerman province, as well as in some other provinces of Iran (Khoramrooz et al. 2023; Vali et al. 2023).

Over 500 species of bacteria are involved in the formation of dental plaque; they are a very complex bacterial community that accumulates in the hard tissues of the oral cavity (Rosan and Lamont 2000). There is no doubt that lactobacilli is one of the most cariogenic bacteria in the oral environment. While it is not the initiator of caries, it plays a crucial role in its progression. Some lactobacilli, such as *Lactobacillus gasseeri*, *L. fermentum*, *L. vaginalis*, and *L. casei*, have been reported to be prevalent at the majority of oral sites, such as saliva, tongue, carious lesions, and dental plaques (Ahirwar, Gupta, and Snehi 2019; Wen et al. 2022). Numerous obligate and facultative anaerobic bacteria predominate in the microbial communities linked to dental caries. Mutans streptococci are the major cariogenic pathogens of tooth decay. Mutans streptococci isolated from dental caries samples are *S. mutans* and *S. sobrinus*. The acidogenic nature of these bacteria allows them to soften teeth's hard tissues by producing short‐chain acids. In addition, the presence of *S. mutans* is believed to be one of the main triggers of dental caries (Abranches et al. 2018; Okada et al. 2005). Bacteria, fluoride, saliva, and sugar are some of the factors that may alter the dynamic flow of demineralization and remineralization in the enamel. During childhood, pediatricians and families can manage these controllable factors to prevent, slow, or delay the progression of the disease (Krol and Whelan 2023).

Snyder's test is one of the routine tests used to determine susceptibility to dental caries by qualitative estimation of acid production of the microbial community of the oral environment. However, it has some limitations; insufficient specificity in identifying certain groups of organisms involved in caries is the main limitation, as this can lead to false positive results (Kunte et al. 2013). Therefore, the availability of other tests, such as measuring the levels of the responsible bacteria (e.g. lactobacilli and *S. mutans*) can assist in pinpointing the caries more precisely. This study aims to integrate Snyder's test, colony counting, and PCR techniques to provide a more comprehensive assessment of the caries activity of *S. mutans* and lactobacilli, which has not been thoroughly explored in the pediatric population aged 5–9 in Kerman province, Iran.

## 2 Materials and Methods

### 2.1 Sample Collection

This cross‐sectional study was executed in the Department of Pediatric Dentistry, School of Dentistry, Kerman University of Medical Sciences, Kerman, Iran between March and Jun 2024. As mentioned, the prevalence of dental caries in the Iranian population is 72.8%. In our study, Formula 1 which was described by (Pourhoseingholi, Vahedi, and Rahimzadeh 2013), was used for the sample size calculation. In this formula, the confidence level is represented by the *Z* statistic (in α = 10% and 90% confidence interval [CI], it is 1.645). The *P* statistic is the expected prevalence (it was 0.728 in our study). The d statistic indicates the precision, or effect size (in relative precision of 10%, it was 0.0728).

(1) $$N=\frac{Z^{2}P\left(1-P\right)}{d^{2}}$$

According to Formula 1, the minimum total sample size (*N*) for conducting the study on dental caries in the Iranian population was calculated to be approximately 101.

The present study involved 120 children (62 [51.67%] boys and 58 [48.33%] girls) aged 5–9 (6.92 ± 1.52) years old. The sampling was carried out by three dental residents under the supervision of a pediatric dentist. Salivary flow and saliva concentration vary within 24 h; for this reason, saliva was collected from all children in the morning between 9:00 and 10:00 a.m and later. Children had not eaten for at least 30 min before sampling. Saliva samples (approximately 2–3 mL) from children who met the eligibility requirements were placed into sterile test tubes with transfer fluid and stored at 10°C–5°C. The tubes were then promptly moved to the laboratory, where they were cultured for a maximum of 3 h. The maximum time of sampling was 1 min, which sometimes reached this maximum time based on the amount of saliva collected.

Three pediatric dentists evaluated the oral and dental status of these children, and then they were categorized into four groups based on dmft index: Group 1 (controls) had a dmft of 0, Group 2 had a dmft of 4–6, Group 3 had a dmft of 7–9, and Group 4 had a dmft of 10–13. Using a questionnaire, parents were asked to provide information about their children's daily habits (including the frequency of brushing their teeth, main meals, and sweet snacks), and parents' educational levels (Table 1). The Research Ethics Committee of Kerman University of Medical Sciences approved the study (IR. KMU. REC.1402.449). Furthermore, a written consent form was obtained from the parents of the children participating in the study.

**Table 1:** Demographic information of the population study.

Features		Males (*n* = 62)				Females (*n* = 58)			
		dmft Groups				dmft Groups			
		G1	G2	G3	G4	G1	G2	G3	G4
**Main meals** (frequency)	2	3	3	1	1	0	5	3	0
	3	11	14	12	9	14	4	17	7
	> 3	0	2	4	2	2	1	4	1
**Sweet snacks** (frequency)	1	6	5	3	5	7	4	7	3
	2	3	8	6	1	5	6	7	4
	3	5	6	5	2	4	0	4	0
	> 3	0	0	3	4	0	0	6	1
**Brushing teeth** (frequency)	0	0	10	9	5	3	3	8	5
	1	12	9	7	6	8	7	12	0
	2	2	0	1	1	5	0	3	0
	3	0	0	0	0	0	0	1	0
**Mothers' educational level**	Low education	1	0	3	2	0	1	4	1
	High school diploma	1	11	10	5	2	6	9	4
	Undergraduate degree	7	6	3	5	8	2	6	2
	Graduate degree	5	2	1	0	6	1	5	1
**Fathers' educational level**	Low education	1	1	6	3	0	1	4	1
	High school diploma	2	11	7	6	2	7	12	5
	Undergraduate degree	6	5	3	2	6	0	4	1
	Graduate degree	5	2	1	1	8	2	4	1

Abbreviations: dmft, decayed missing and filled (primary) teeth; G, Group.

#### 2.1.1 Inclusion Criteria

a. Children aged 5–9 years old who were originally from Kerman Province and referred to the Department of Pediatric Dentistry.
b. The Children had no inflammatory, oral, bacterial, systematic, or other diseases that influence saliva secretion. In addition, they had not taken any antibiotics for at least 14 days before sampling.
c. Children's saliva was collected without the use of saliva stimulants.
d. Children and parents were willing to participate in the study voluntarily.

#### 2.1.2 Exclusion Criteria

a. Children with dmft indices 1–3 and over 13. The main reason is that there are very few cases with a dmft index over 13 in the pediatric population; also, it is hard to find substantial variations in microbiota in the dmft indices 1–3.
b. Individuals who did not meet the age group requirements or were not residents of Kerman Province.
c. Children whose parents had not signed the consent form.
d. Incomplete responses to the questionnaire.
e. To prevent any potential bias in the colony counting of bacteria, children who had exfoliation of teeth were excluded from the sampling.

### 2.2 Caries Activity

#### 2.2.1 Snyder's Test

B.C.G‐Dextrose Agar (Quelab Company, Montreal, QC, Canada) was used as Snyder's test medium to qualitatively determine the caries activity of *S. mutans*, lactobacilli, and some other acidogenic microbes involved in dental caries. The medium contains the following components: peptone (20 g/L), dextrose (glucose [20 g/L]), sodium chloride (5 g/L), bromocresol green (0.02 g/L), agar (20 g/L). The final pH of the medium at 25°C was 4.8 ± 0.2. Test tubes of Snyder's test were prepared according to the manufacturer's instructions: first, 65.02 g of the medium powder was suspended in 1000 mL of distilled water. Then to completely dissolve the medium, it was heated till boiling. After that, 10 mL amounts of medium was dispensed in each test tube. Subsequently, the test tubes were sterilized by autoclaving at 121°C for 15 min. Finally, the test tubes were cooled at an upright position.

In the current study for conducting Snyder's test, 100 µL of saliva was added to each test tube. Incubation was then performed on these test tubes for 24, 48, and 72 h at 37°C. When cariogenic bacteria exist in the saliva, glucose is fermented and lactic acid is produced, accordingly reducing the pH to approximately 4.4 in the medium. The severity of caries is characterized by the rate at which the color changes from green to yellow. Depending on the situation, one of the four following patterns may occur: (1) complete yellowing within 24 h represents a “marked susceptibility” to creating dental caries; (2) yellowing up to 48 h represents a “definitive susceptibility”; (3) yellowing up to 72 h represents a “limited susceptibility”; and (4) no change in color (green) occurs within 72 h, indicating a “negative susceptibility” (Ali et al. 1998; Ramesh et al. 2013; Snyder 1940).

#### 2.2.2 Colony Counting of *S. mutans*

In this study, the Mitis Salivarius Agar Base (Quelab Company, Montreal, QC, Canada) was used as a selective medium for isolation and counting colonies of *S. mutans*. The medium consists of these components: casein enzymic hydrolysate (15 g/L), peptic digest of animal tissue (5 g/L), dextrose (1 g/L), sucrose (50 g/L), dipotassium phosphate (4 g/L), trypan blue (0.075 g/L), crystal violet (0.0008 g/L), and agar (15 g/L). The medium's final pH was 7 ± 0.2 at 25°C.

The manufacturer's instructions were pursued to prepare the medium: The 90.07 g of the medium powder was suspended in 1000 mL of distilled water. Complete dissolution of the medium was obtained by heating it to boiling. Sterilization of the medium was granted using autoclaving at 121°C for 15 min. Afterward, the medium was cooled to 50°C–55°C and then 1 mL of sterile 1% potassium tellurite solution was added to the medium (after this step, the medium should not be reheated). Ultimately, the medium was mixed well and poured into Petri plates.

For conducting colony counting of *S. mutans*, 0.2 mL of saliva was added to 1.8 distilled water in a 2 mL tube (dilution of 10^−1^), then 0.2 of the sample from the tube was added to 1.8 mL of distilled water in a new 2 mL tube (dilution of 10^−2^). The process was continued till the serial dilution reached 10^−5^. From this dilution (10^−5^), 1 mL of sample was inserted into the Petri plate. After 48 h of incubation at 35°C, colonies of *S. mutans* appeared in the medium, and they were counted using a naked eye.

The count of S. mutans was estimated using the number of times of serial dilution multiplied by the number of colony‐forming units (CFU); the output was demonstrated as CFU/mL of saliva (Ademe, Admassu, and Balakrishnan 2020). As the serial dilution of the saliva was 5, the colonies of *S. mutants* were presented based on 10^5^ CFU/mL.

#### 2.2.3 Colony Counting of Lactobacilli

To isolate and colony count of lactobacilli, the medium of de Man, Rogosa, and Sharpe (MRS) Agar (Quelab Company, Montreal, QC, Canada) was utilized (de Man, Rogosa, and Sharpe 1960). The composition of the medium was proteose peptone (10 g/L), beef extract (8 g/L), yeast extract (4 g/L), dextrose (20 g/L), polysorbate 80 (1 g/L), ammonium citrate (2 g/L), Sodium acetate (5 g/L), magnesium sulfate (0.2 g/L), manganese sulfate (0.05 g/L), dipotassium phosphate (2 g/L), and agar (14 g/L); the pH of the medium at 25°C was 6.2 ± 0.2.

To prepare the culture medium, the instructions of the manufacturer were followed: In 1000 mL distilled water, 64 g of medium powder was suspended and heated to dissolve the medium completely. Then the medium was distributed in Petri plates and was sterilized by autoclaving at 121°C for 15 min. For isolation and colony counting of lactobacilli, serial dilution of saliva (10^−5^) which was above described for *S. mutans* was used; the amount of 1 mL of this serial dilution of saliva was inserted into the MRS agar medium. Plates were incubated anaerobically using Microbiology Anaerocult C (Merck Company, United States) in anaerobic jars for 48 h at 37°C. After that, colonies of lactobacilli appeared on the plates and then they were counted with the naked eye. The strategy that was described for calculating the colony counting of *S. mutans* was also performed for lactobacilli (Ademe, Admassu, and Balakrishnan 2020); hence, the 10^5^ CFU/mL was also used for the count of lactobacilli.

#### 2.2.4 Molecular Identification

DNA was extracted from children's saliva using a standard protocol (Goode et al. 2014). Primers used for detection of the *gftB* gene in *S. mutans* were: “F5ʹ‐ ACTACACTTTCGGGTGGCTTGG‐3ʹ” and “R5ʹ‐CAGTATAAGCGCCAGTTTCATC‐3ʹ” (Franco e Franco et al. 2007). Furthermore, the *16S rRNA* gene was used for the detection of lactobacilli with these primers: “F5ʹ‐CATTTGGAAACAGATGCTAATACC‐3ʹ, and R5ʹ‐GTCCATTGTGGAAGATTCCC‐3ʹ” (Pahumunto et al. 2019). Then using PCR assay, the presence of *S. mutans* and lactobacilli was evaluated as mentioned as follows. Each tube of PCR was prepared with these components: 10 µL of Taq DNA Polymerase 2x Master Mix RED (Ampliqon Co., Denmark), 150 ng of extracted DNA, 10 pmol/µL of each forward and reverse primers, and the final volume of reaction tube was reached to 20 µL with distilled water. Also, for negative control, distilled water was used instead of DNA, and for positive control, purified genomic DNA of *S. mutans* or lactobacilli was used instead of DNA of saliva. The steps of the PCR program were as follows: (I) 95°C for 5 min; (II) 40 (for *16S rRNA*)/45 (for *gftB*) cycles of 95°C for 30 s, 54°C for 45 s, and 72°C for 45 s; and (III), a final extension phase was applied on 72°C for 5 min. Ultimately, PCR products were run on a 2% agarose gel electrophoresis together with a DNA ladder, negative control, and positive control.

### 2.3 Statistical Analysis

**Figure 1:**
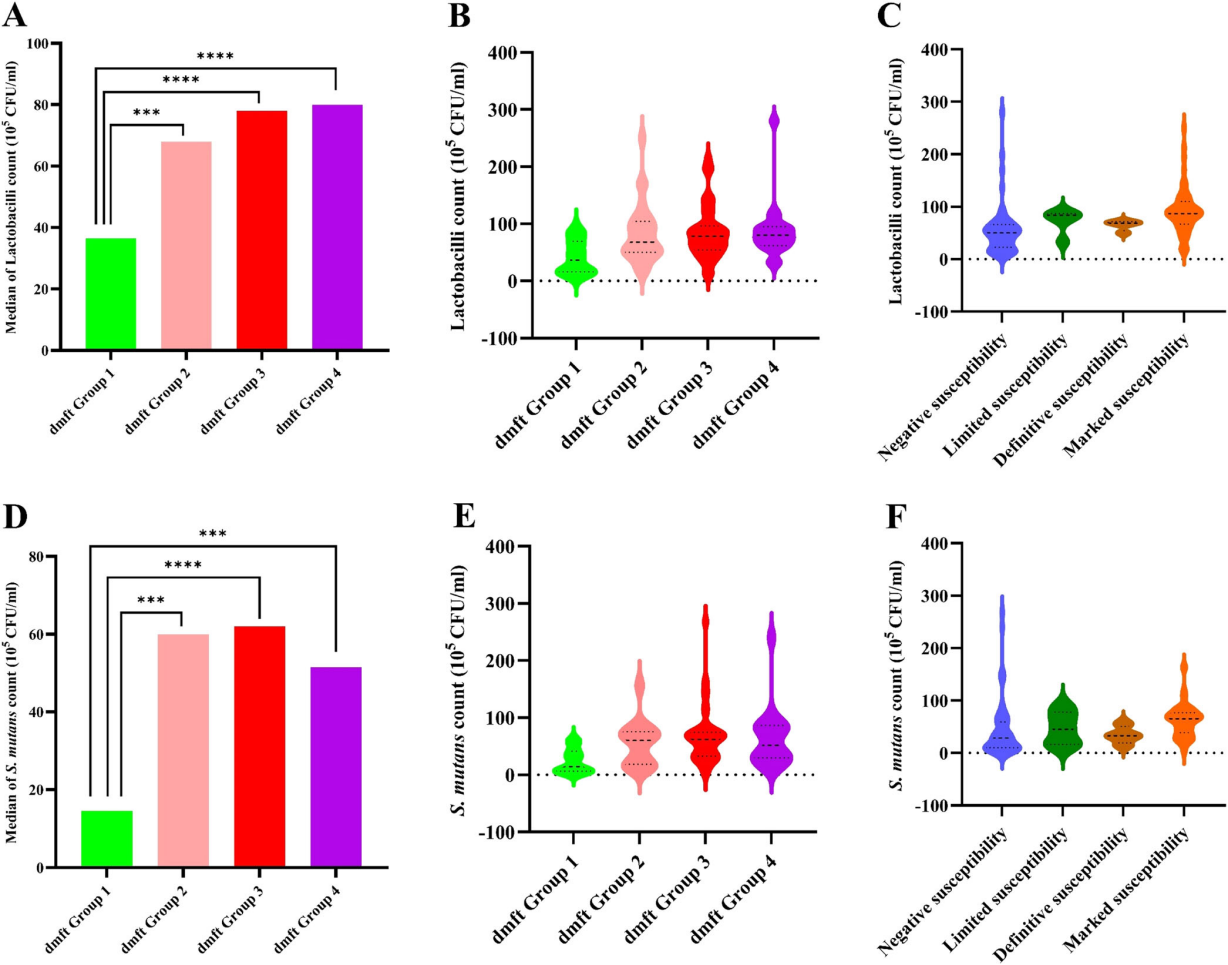
The significance and distribution of lactobacilli and *S. mutans* counts within the dmft Groups and the Snyder's test. Comparison of the median counts of lactobacilli (A) and *S. mutans* (D) in the dmft Group 1 with Groups 2–4 using the Mann–Whitney test; there are significant differences in dmft Group 1 with other gmft Groups for lactobacili and *S. mutans*. Violin plots of the distribution of lactobacilli (B) and *S. mutans* counts (E) in the dmft Groups; these violin plots show that lactobilli and *S. mutans* have low levels in dmft Group 1 in comparisons of other dmft Groups. Violin plots of lactobacilli (C) and *S. mutans* counts (F) in the results of Snyder's test. These violin plots demonstrate that the low level of lactobacilli is associated with negative susceptibility conditions of Snyder's test; however, the low level of *S. mutans* is observed in marked susceptibility (Table S3). CFU, colony forming unit; *S. mutans*, *Streptococcus mutans*; ***significant at *p* < 0.001; *****significant at *p* < 0.0001.

All statistical tests were performed utilizing SPSS version 27.0 (SPSS Inc., Chicago, USA), MedCalc version 22.021 (MedCalc Software Ltd., Ostend, Belgium) (for calculating odds ratio), and GraphPad Prism version 10.2.3 (GraphPad Software Inc, Boston, MA, USA, for generating bar chart and violin charts; Figure 1] with a *p* value < 0.05 threshold. The following statistical tests were used in the study.

The Kolmogorov–Smirnov test, which is a robust normality test for sample sizes over 50 (Mishra et al. 2019), was performed to evaluate the normal distribution of numerical data, including children's age, *S. mutans* count, and lactobacilli count; the outputs of the test were considered for choosing parametric or nonparametric tests. To determine the differences between a numerical variable and a dichotomous variable, the nonparametric Mann–Whitney U test was utilized; also, the effect size for this test was calculated using Formula 2.

(2) $$\text{Cohen}\prime s\;d=\frac{\left|Z\;\text{score}\right|}{\sqrt{N}}$$

The nonparametric Kruskal–Wallis test was used to find differences between a numerical variable and a multi‐state (three or more) variable. The chi‐square test was employed to compare two nominal/ordinal variables. The nonparametric Spearman's Correlation Coefficient test was used to determine the possible correlation among numerical variables. Scheffe's post hoc test was utilized for evaluating differences in bacterial count among dmft groups.

## 3 Results

### 3.1 The Status of Normal Distribution

In the present study, quantitative data (e.g., children's age and counts of *S. mutans* and lactobacilli) were checked using the Kolmogorov–Smirnov test to assess their normality. Since the outputs revealed that these variables were not normally distributed (age: *N* = 120, mean = 6.93, SD = 1.52, *p* < 0.001; *S. mutans* count: *N* = 120, mean = 52.52, SD = 44.33, *p* < 0.001; and lactobacilli count: *N* = 120, mean = 73.78, SD = 47.95, *p* < 0.001), non‐parametric tests should be applied for subsequent statistical analyses. Since the number of bacteria in a population varies from person to person, it is not out of the question that the population will not follow a normal distribution.

### 3.2 Assessment of the Roles of Bacterial Counts and the Age of the Children in Dental Caries

The Mann–Whitney U test was used to evaluate significant differences between the bacterial count and the age of the children with bacterial PCR and the gender of the children (Table 2).

**Table 2:** Comparison of *S. mutans* count, lactobacilli count, and age in gender, *S. mutans* PCR (*gftB* gene), and lactobacilli PCR (*16S rRNA* gene) using Mann–Whitney *U* test.

	Variable status	*n*	Mean Rank	Sum of Rank	*U*	*Z*	Cohen's *d*	*p* value
*S*. *mutans* count								
*S. mutans* PCR	*gftB* negative	61	41.77	2548	657	−5.999	0.547	< 0.001*
	*gftB* positive	59	79.86	4712				
Lactobacilli PCR	*16S rRNA* negative	16	25.75	412	276	−4.293	0.392	< 0.001*
	*16S rRNA* positive	104	65.85	6848				
Gender	Male	62	60.97	3780	1769	−0.152	0.014	0.879
	Female	58	60	3480				
Lactobacilli count								
*S. mutans* PCR	*gftB* negative	61	44.83	2734.5	843.5	−5.02	0.458	< 0.001*
	*gftB* positive	59	76.7	4525.5				
Lactobacilli PCR	*16S rRNA* negative	16	24.88	398	262	−4.402	0.402	< 0.001*
	*16S rRNA* positive	104	65.98	6862				
Gender	Male	62	59.57	3693.5	1740.5	−0.302	0.028	0.763
	Female	58	61.49	3566.5				
Age								
*S. mutans* PCR	*gftB* negative	61	53.03	3235	1344	−2.45	0.224	0.014*
	*gftB* positive	59	68.22	4025				
Lactobacilli PCR	*16S rRNA* negative	16	56.03	896.5	760.5	−0.566	0.052	0.572
	*16S rRNA* positive	104	61.19	6363.5				
Gender	Male	62	60.73	3765.5	1783.5	−0.078	0.007	0.938
	Female	58	60.25	3494.5				

Abbreviations: *S. mutans*, *Streptococcus mutans*. d statistic indicates the precision, or effect size.

* Statistically significant.

In addition, the association between children's age and bacterial counts with children's habits and the educational level of their parents was measured using the Kruskal–Wallis test (Table 3).

**Table 3:** Evaluation of the relationship between bacterial counts and children's age with children's habits and parents' educational level using the Kruskal–Wallis test.

	Lactobacilli count (*N* = 120)			*S. mutans* count (*N* = 120)			Age (*N* = 120)		
	df	*H*	*p* value	*df*	*H*	*p* value	*df*	H	*p* value
Brushing teeth	3	7.207	0.066	3	2.043	0.564	3	0.427	0.935
Sweet snacks	3	5.726	0.126	3	7.271	0.064	3	11.367	0.01*
Main meals	2	0.83	0.66	2	2.346	0.309	2	3.552	0.169
Mothers' educational levels	3	9.189	< 0.001*	3	9.189	0.027*	3	5.196	0.158
Fathers' educational levels	3	18.457	< 0.001*	3	20.46	< 0.001*	3	14.437	0.002*
dmft groups	3	22.436	< 0.001*	3	25.998	< 0.001*	3	6	0.112
Snyder's test	3	32.472	< 0.001*	3	19.549	< 0.001*	3	5.374	0.146

Abbreviations: *df*, degrees of freedom; *H*, Kruskal–Wallis *H*; dmft, decayed missing and filled (primary) teeth; *S. mutans*, *Streptococcus mutans*.

* Statistically significant.

In Figure 1, the counts of lactobacilli (Figure 1A) and *S. mutans* (Figure 1D) in dmft Group 1 are compared with dmft Groups 2, 3, and 4 using the Mann–Whitney *U* test, and the bar chart–based median of counts is created. This test showed the lactobacilli levels were significantly different in dmft Group 1 with Group 2 (Total *N* = 59, Mean Rank; Group 1 = 22.32, Group 2 = 37.95; *U* = 204.5, *Z* = −3.498, Cohen's *d* = 0.455, *p* = 0.0003), Group 1 with Group 3 (Total *N* = 71, Mean Rank; Group 1 = 24.3, Group 3 = 44.56; *U* = 264, *Z* = −4.087, Cohen's *d* = 0.485, *p* < 0.0001), and Group 1 with Group 4 (Total *N* = 50, Mean Rank; Group 1 = 19.1, Group 4 = 35.1; *U* = 108, *Z* = −3.804, Cohen's *d* = 0.538, *p* < 0.0001). The *S. mutans* levels were also notably different in dmft Group 1 with Group 2 (Total *N* = 59, Mean Rank; Group 1 = 21.93, Group 2 = 38.34; *U* = 193, *Z* = −3.671, Cohen's *d* = 0.478, *p* = 0.0002), Group 1 with Group 3 (Total *N* = 71, Mean Rank; Group 1 = 22.42, Group 3 = 45.94; *U* = 207.5, *Z* = −4.745, Cohen's *d* = 0.563, *p* < 0.0001), and Group 1 with Group 4 (Total N = 50, Mean Rank; Group 1 = 19.5, Group 4 = 34.5; *U* = 120, *Z* = −3.566, Cohen's *d* = 0.504, *p* = 0.0002). The distribution status of lactobacilli (Figure 1B) and *S. mutans* counts (Figure 1E) in all dmft Groups is also shown in the violin plots. In addition, the counts of lactobacilli (Figure 1C) and *S. mutans* (Figure 1F) in the results of Snyder's test are also specified in the violin plots.

**Figure 2:**
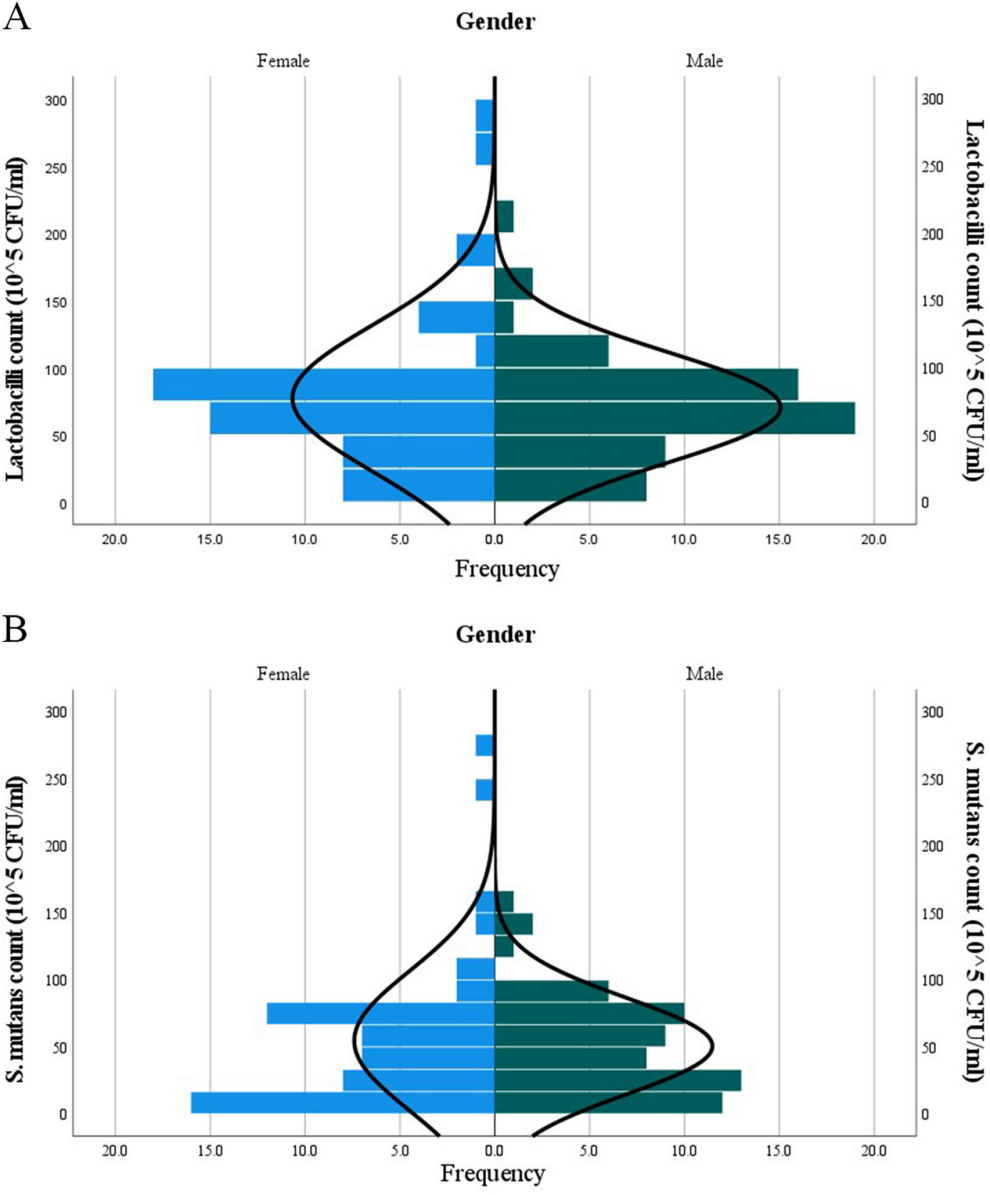
Pyramid plots of bacterial counts and gender of children. (A) lactobacilli count and (B) *S. mutans* count. Data in the Y‐axis demonstrate colony counts of both bacteria based on gender. The X‐axis shows the frequency of children in each gender. There are no significant differences between colony counts of bacteria within gender, according to the Mann–Whitney *U* test (refer to Table 2): lactobacilli: *U* = 1740.5, Cohen's *d* = 0.028, *p* = 0.763; *S. mutans*: *U* = 1769, Cohen's *d* = 0.014, *p* = 0.879. CFU, colony forming unit; *S. mutans*, *Streptococcus mutans*.

The distributions of lactobacilli and *S. mutans* counts in children's gender are depicted in Figure 2.

### 3.3 Findings of PCR and Snyder's Test

In Table 4, the roles of bacterial PCR, Snyder's test, and dmft groups in children's habitual variables and parents' educational level were determined using the chi‐square test.

**Table 4:** The association of lactobacilli PCR, *S. mutans* PCR, Snyder's test, and dmft Groups with each other and with demographic characteristics using the chi‐square test.

	Lactobacilli PCR (*n* = 120)			*S. mutans* PCR (*n* = 120)			Snyder's test (*n* = 120)			dmft Groups (*n* = 120)		
	*df*	*χ* ^*2*^	*p* value	*df*	χ^2^	*p* value	*df*	*χ* ^*2*^	*p* value	*df*	*χ* ^*2*^	*p* value
**Gender**	1	0.294	< 0.001*	1	0.463	0.496	3	1.908	0.592	3	4.794	0.188
Brushing teeth	3	5.396	0.145	3	1.581	0.664	9	10.102	0.342	9	18.895	0.026*
Sweet snacks	3	5.213	0.157	3	12.113	0.007*	9	15.879	0.069	9	20.868	0.013*
Main meals	2	2.976	0.226	2	2.013	0.336	6	2.034	0.917	6	9.583	0.143
Mothers’ educational level	3	11.042	0.011*	3	1.072	0.784	9	7.711	0.563	9	28.539	< 0.001*
Fathers’ educational level	3	9.226	0.026*	3	5.748	0.125	9	6.377	0.702	9	32.817	< 0.001*
dmft Groups	3	13.911	0.003*	3	4.387	0.223	9	9.155	0.423	—	—	—
Lactobacilli PCR	—	—	—	1	13.6	< 0.001*	3	7.428	0.059	3	13.911	0.003*
*S. mutans* PCR	1	13.6	< 0.001*	—	—	—	3	39.965	< 0.001*	3	0.223	4.387
Snyder's test	3	7.428	0.059	3	39.965	< 0.001*	—	—	—	9	9.155	0.423

Abbreviations: χ2, Chi‐Square; *df*, degrees of freedom; dmft, decayed missing and filled (primary) teeth; *S. mutans*, *Streptococcus mutans*.

* Statistically significant.

**Figure 3:**
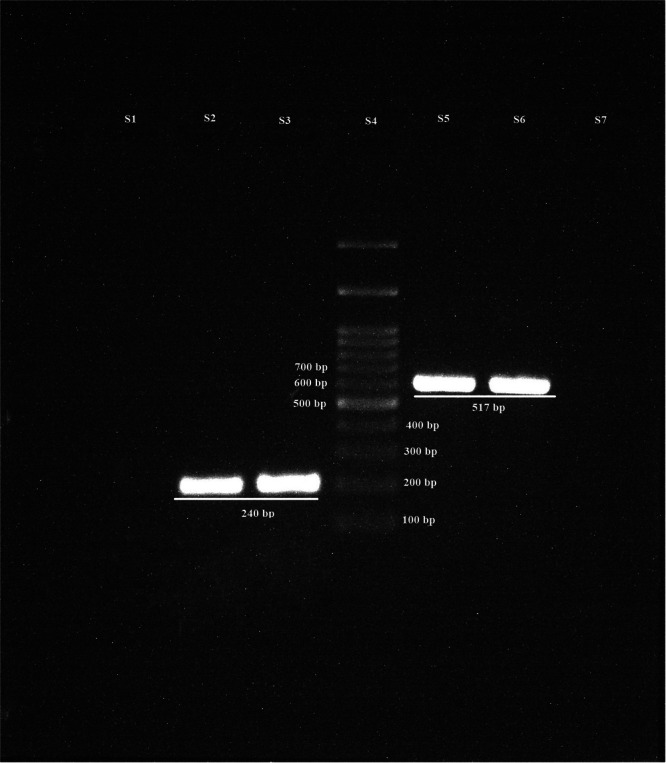
Two percent agarose gel electrophoresis of PCR products for detecting lactobacilli (*16S rRNA* gene with 240 bp length) and *S. mutans* (*gftB* gene with 517 bp length). S1, negative control of lactobacilli; S2, positive control of lactobacilli; S3, a child with positive PCR for lactobacilli; S4, DNA size marker; S5, a child with positive PCR for *S. mutans*; S6, positive control of *S. mutans*; S7, negative control of *S. mutans*; bp, base pair; *S. mutans*, *Streptococcus mutans*.

The PCR products of lactobacilli and *S. mutans* were run on 2% agarose gel electrophoresis (Figure 3).

The number of children with positive PCR results of lactobacilli was 104/120 (86.67%), and the lactobacilli count was 80.11 ± 47.37 (10^5^ CFU/mL) in them. In children with negative PCR results of lactobacilli, these were 16/120 (13.33%) and 32.63 ± 25.17 (10^5^ CFU/mL), respectively. In addition, the positive PCR results of *S. mutans* in the affected children were 59/120 (49.17%) and the *S. mutans* count was 69.12 ± 34.74 (10^5^ CFU/mL); for positive PCR results of the bacterium, these were 61/120 (50.83%) and 36.46 ± 46.87 (10^5^ CFU/mL), respectively.

Also, children who had positive PCR of lactobacilli and marked susceptibility (the highest grade of acidity determined by Snyder's test) were 54/120 (45%) and the lactobacilli count was 93.15 ± 44.76 (10^5^ CFU/mL). Moreover, children with positive PCR of *S. mutans* and marked susceptibility were 45/120 (37.5%) and the *S. mutans* count was 69.15 ± 29.7 (10^5^ CFU/mL).

Also, 44/120 (36.67%) children had positive PCRs of lactobacilli and marked susceptibility to acid production; they had 94.66 ± 44.30 (10^5^ CFU/mL) for lactobacilli count and 69.18 ± 30.05 (10^5^ CFU/mL) for *S. mutans* count.

The frequency of Snyder's test and lactobacilli and *S. mutans* PCRs in dmft Groups is shown in Table S1. The frequency of lactobacilli and *S. mutans* counts in dmft Groups is presented in Table S2. The frequency of lactobacilli and *S. mutans* counts in the results of Snyder's test is provided in Table S3. The frequency of lactobacilli and *S. mutans* PCRs in the results of Snyder's test is demonstrated in Table S4. The frequencies of children's daily habits and parents’ educational level in the PCRs and the counts of lactobacilli and *S. mutans* are supplied in Table S5. The frequencies of children's daily habits and parents' educational level in the results of Snyder's test are provided in Table S6.

Specifically, in Table S7, the relationship of each dmft Group with other dmft groups is examined using Scheffe's post hoc test. Based on the outputs of Scheffe's test, there are significant differences between Group 1 with Groups 2–4 for both *S. mutans* and lactobacilli counts. However, there was no significant relationship between these bacterial counts within dmft Groups 2–4.

Predicting the relationship between lactobacilli count, *S. mutans* count, and age with each other within dmft groups and in total using Spearman's Correlation Coefficient test is provided in Table S8. The findings of Table S8 revealed that lactobacilli count, *S. mutans* count, and age had significant positive correlations with one another. In dmft Group 1, correlation between lactobacilli count and *S. mutans* count was positive (*n* = 30, *r* = 0.848, *p* < 0.001), in dmft Groups 2–4 they also were positive (*n* = 90, *r* = 0.514, *p* < 0.001); moreover, in total participants (dmft Groups 1–4), they were positive (*N* = 120, *r* = 0.662, *p* < 0.001).

One of the key results is that increasing the children's age is a contributing factor to the increasing lactobacilli (*N* = 120, *r* = 0.389, *p* < 0.001) and *S. mutans* count (*N* = 120, *r* = 0.352, *p* < 0.001) in dental caries.

As mentioned earlier in Table 3, there found significant differences between the count of lactobacilli (*N* = 120, *df *= 3, *H* = 22.436, *p* < 0.001) and *S. mutans* (*N* = 120, *df *= 3, *H* = 25.998, *p* < 0.001) with dmft Groups.

Additionally, for evaluation of the outcomes of bacterial PCR within dmft Groups, odds ratio (OR) was estimated within dmft Groups (Table S9). As shown in Table S9, lactobacilli PCR had a high significant risk in dmft Group 1 with dmft Groups 2 (OR = 7.816, *p* = 0.013), 3 (OR = 7.333, *p* = 0.005), and 4 (OR = 11, *p* = 0.028). However, the OR of *S. mutans* PCR was only significantly higher in dmft Group 3 compared to dmft Group 1 (OR = 2.699, *p* = 0.045).

## 4 Discussion

Our study aimed to investigate the effect of Snyder's test and the levels and PCRs of *S. mutans* and lactobacilli in children with (dmft Groups 2–4) and without (dmft Group 1) dental caries. Neither age nor the counts of *S. mutans* and lactobacilli did not differ significantly by gender (Table 2). Likewise, age did not differ with lactobacilli PCR but differed with *S. mutans* PCR. However, the *S. mutans* and lactobacilli counts had significant differences with their PCR results. A study executed by Lee et al. determined the caries activity of *S. mutans* using PCR and found that the portion of *S. mutans* identified in the plaque sample is 56.8%, and in the saliva sample it is 79.7%. Also, they reported that among participants (children, adolescents, and adults), adolescents had the highest levels of *S. mutans* in saliva, and adults had the highest levels in plaque samples (Lee et al. 2019).

Table 3 demonstrated that no significant differences were found between the counts of lactobacilli and *S. mutans* with brushing teeth, sweet snacks, and main meals. However, they had a remarkable association with mothers' and fathers' educational levels, dmft groups, and Snyder's test. In addition, age was related to sweet snacks and fathers' educational levels, but it was not related to other variables. A study on a group of Indian schoolchildren found that the *S. mutans* count, frequency of food intake, and food content had notable roles in the risk of dental caries (Jagan et al. 2021).

According to Table 4, lactobacilli PCR had significant differences with gender, mothers' and fathers' educational levels, dmft Groups, and *S. mutans* PCR; but had no differences with brushing teeth, sweet snacks, main meals, and Snyder's test. *S. mutans* PCR had significant differences with sweet snacks, lactobacilli PCR, and Snyder's test. Surprisingly, Snyder's test had only a significant difference with *S. mutans* PCR.

The dmft Groups had significant differences with brushing teeth, sweet snacks, mothers' and fathers' educational levels, and lactobacilli PCR. A study conducted by Sajadi et al. on Iranian children aged 3–6 years in Kerman, Iran, showed that the dmft index had significant difference with the mother's educational level, eating sweets and biscuits, toothbrush use, and children's age. However, they did not find any difference between the dmft index and gender (Sajadi et al. 2018). The results of Sajadi's study are very similar to our study, but they did not assess the impact of the number of main meals that children eat per day; in our study, main meals did not differ between the dmft groups (Table 4). One study reported that low levels of *S. mutans* in individuals with high levels of dental caries could be a result of having special strains of *S. mutans* (Toi, Cleaton‐Jones, and Daya 1999). In our study, we did not isolate and differentiate these strains of *S. mutans*.

Host–microbiota–diet interactions may be manageable to reduce the risk of dental caries. The prevention strategies should be applied to children's behavioral and dietary habits to reduce the risk of oral and dental problems (Anil and Anand 2017). Our study found a positive association between increasing children's age and increasing the number of lactobacilli and *S. mutans* (Table S8). Hence, controlling host–microbiota–diet interactions should be a primary strategy, at least in younger children.

Some studies reported that significant correlations exist between oral lactobacilli counts and the severity of dental caries (Ademe, Admassu, and Balakrishnan 2020; Piwat et al. 2010). However, a study by Eşian et al. in Romania did not find a correlation between lactobacilli counts and dental caries, but they found a correlation between *S. mutans* counts and dental caries (Eşian et al. 2017). Because of *S. mutans*' capacity to form biofilm by synthesizing glucan, ability to produce acid, and acid tolerance, a high concentration of S. *mutans* has been correlated to dental caries (Gao et al. 2024). Probiotic lactobacilli have been shown to reduce and/or inhibit the caries activity of *S. mutans* in dental caries which could be used as a suitable therapy, especially for at‐risk children (Wen et al. 2022). Our study found significant positive correlations between *S. mutans* count and lactobacilli count in dmft Groups. In contrast with our study, Sounah et al. used real‐time PCR to assess the microbial community involved in dental caries in adult Yemeni people, surprisingly they did not find any relation between *S. mutans* and lactobacilli levels with DMFT index (Sounah and Madfa 2020). Also, a study in Iran by Najafi et al. showed that there is no correlation between lactobacilli counts and *S. mutans* count in patients with dental caries (Najafi et al. 2022).

A systematic review study reported that while lactobacilli are not inherently efficient at adhering to tooth surfaces compared to their cariogenic collaborator, mutans streptococci; therefore, their colonization potential is markedly improved in the presence of initial colonizers like *S. mutans*; hence, lactobacilli are associated with advanced dental caries regardless of age (Wen et al. 2022). Our study also had a similar conclusion because the average of the lactobacilli count in dmft Group 1 is about 97.01% lower than in dmft Group 2, 100.02% in dmft Group 3, and 100.09% in dmft Group 4. The average of the *S. mutans* count in dmft Group 1 was 129.09% lower than in dmft Group 2, 175.05% in dmft Group 3, and 159.84% in dmft Group 4 (Table S2). A randomized clinical trial on children 3–9 years old in Kerman, Iran, showed that Biodentine and formocresol pulpotomy techniques may be suitable as a good treatment for children suffering from primary molars (Gisour et al. 2023).

One study identified 18 phylotypes of lactobacilli in dental caries by *16S rRNA* sequencing and phylogenetic analysis; they also measured the concentration of lactobacilli using real‐time PCR and observed that it was about 34 times higher than when measuring CFU (Byun et al. 2004). A new strategy for reducing the levels of *S. mutans* and lactobacilli is the use of kidodent and probiotic mouth rinse (Bolla et al. 2023).

It should be noted that some limitations were encountered during our study, including the refusal of some parents to complete the questionnaire and the refusal of some of them to allow their children to undergo dental examinations. As an additional limitation, this study was executed exclusively in Kerman Province, so the results may not be generalized to other Iranian provinces due to their localized nature. The authors recommend that further studies with a large sample size should be commissioned. Nowadays, even though the incidence of dental caries in children has decreased globally compared to past decades, there is still a need for public dental screening policies to prevent dental caries in children.

## 5 Conclusion

There were strong correlations between levels of *S. mutans* and lactobacilli which can accelerate the dental caries process in children; this microbial level can even be strengthened by raising the age of the children. Furthermore, the positive PCR of them was related to the deterioration of tooth decay. Our study proposes that the combination of Snyder's test with PCR and colony counting of *S. mutans* and lactobacilli could serve as a cost‐effective tool for early caries detection in clinical settings. Understanding the precise roles played by these bacteria in childhood dental caries will require more research. The results of our research could be useful to microbiologists, molecular pathologists, healthcare professionals, pediatric dentists, and other related fields dealing with oral and dental problems.

## Author Contributions

Project administration: Marzieh Danaei and Raziyeh Shojaeipour. Supervisions: Marzieh Danaei, Raziyeh Shojaeipour, and Hamidreza Poureslami. Conceptualization: Marzieh Danaei, Raziyeh Shojaeipour, Hamidreza Poureslami, Fatemeh Sadat Sajadi, Elham Farokh Gisour, Fatemeh Jahanimoghadam, and Milad Mollaali. Funding acquisition: Marzieh Danaei. Resources: Marzieh Danaei. Writing manuscript draft: Milad Mollaali. Critical reviewing and editing of final manuscript: Raziyeh Shojaeipour, Fatemeh Sadat Sajadi, Marzieh Danaei, Hamidreza Poureslami, Fatemeh Jahanimoghadam, Elham Farokh Gisour, and Milad Mollaali. Investigations and sample collection: Marzieh Danaei, Vida Fakharmohialdini, Aida Gholampour, Mehrnaz Foroudisefat, Arezoo Mirshekari, Milad Mollaali, Hamidreza Poureslami, Elham Farokh Gisour, Fatemeh Jahanimoghadam, and Raziyeh Shojaeipour. Methodology: Marzieh Danaei, Raziyeh Shojaeipour, Hamidreza Poureslami, Fatemeh Sadat Sajadi, Elham Farokh Gisour, Fatemeh Jahanimoghadam, Milad Mollaali, Vida Fakharmohialdini, Aida Gholampour, Mehrnaz Foroudisefat, and Arezoo Mirshekari. Formal analysis: Milad Mollaali and Marzieh Danaei. Software: Milad Mollaali. Data curation: Milad Mollaali, Raziyeh Shojaeipour, and Marzieh Danaei. Visualization: Milad Mollaali. Validation: Hamidreza Poureslami, Fatemeh Sadat Sajadi, Raziyeh Shojaeipour, Marzieh Danaei, Elham Farokh Gisour, Fatemeh Jahanimoghadam and Milad Mollaali. Approving manuscript contents: all authors.

## Ethics Statement

The Research Ethics Committee of Kerman University of Medical Sciences approved the study (IR. KMU. REC.1402.449).

## Consent

A written consent form was obtained from the parents of the children participating in the study.

## Conflicts of Interest

The authors declare no conflicts of interest.

## Supporting information

Supporting information.

## Data Availability

The data that support the findings of this study are available on request from the corresponding author. The data are not publicly available due to privacy or ethical restrictions. The data that support the findings of this study are available from the corresponding author upon reasonable request and with permission from the Research Ethics Committee of Kerman University of Medical Sciences.
