# Supplementary material for: Molecular and Microbial Detections of Streptococcus mutans and Lactobacilli in Dental Caries: An Experimental Study on Iranian Children Aged 5–9
Source: Clin Exp Dent Res. 2024 Dec 12;10(6):e70039. doi: 10.1002/cre2.70039 (PMC11636308; doi:10.1002/cre2.70039)
Supplement: Supplementary file 1 — Supporting information. [file CRE2-10-e70039-s001.docx]

**Supplementary material**

**Molecular and microbial detections of *Streptococcus mutans* and lactobacilli in dental caries: An experimental study on Iranian children aged 5 to 9**

Running Title: Caries activity of *S. mutans* and lactobacilli in children

Marzieh Danaei ^1^, Milad Mollaali ^1,2^, Vida Fakharmohialdini ^1^, Hamidreza Poureslami ^3^, Fatemeh Sadat Sajadi ^3^, Elham Farokh Gisour ^3^, Fatemeh Jahanimoghadam ^4^, Aida Gholampour ^5^, Mehrnaz Foroudisefat ^5^, Arezoo Mirshekari ^5^, Raziyeh Shojaeipour ^3,*^

^1^ *Dana Gene Pajoohan Karmania Company, Member of Iran High-Tech Laboratory Network, Kerman, Iran*

^2^ *Department of Biology, Faculty of Science, University of Sistan and Baluchestan, Zahedan, Iran*

^3^ *Oral and Dental Diseases Research Center, Department of Pediatrics Dentistry, School of Dentistry, Kerman University of Medical Sciences, Kerman, Iran*

^4^ *Social Determinants on Oral Health Research Center, Kerman University of Medical Sciences, Kerman, Iran*

^5^ *Department of Pediatrics Dentistry, School of Dentistry, Kerman University of Medical Sciences, Kerman, Iran*

** Corresponding author: Raziyeh Shojaeipour, E-mail: shojaeipour@kmu.ac.ir, Tel: +98(34)32119028*

**Table S1.** The frequency of Snyder’s test and lactobacilli and *S. mutans* PCRs in dmft Groups.

|  | **dmft Groups** (N=120) | | | |
| --- | --- | --- | --- | --- |
|  | **Group 1** (n=30) | **Group 2** (n=29) | **Group 3** (n=41) | **Group 4** (n=20) |
|  | Count (%) | Count (%) | Count (%) | Count (%) |
| **Lactobacilli PCR** |  |  |  |  |
| Negative | 10 (8.33) | 2 (1.67) | 3 (2.5) | 1 (0.83) |
| Positive | 20 (16.67) | 27 (22.5) | 38 (31.67) | 19 (15.83) |
| ***S. mutans* PCR** |  |  |  |  |
| Negative | 19 (15.83) | 16 (13.33) | 16 (13.33) | 10 (8.33) |
| Positive | 11 (9.17) | 13 (10.83) | 25 (20.83) | 10 (8.33) |
| **Snyder's test** |  |  |  |  |
| Negative susceptibility | 17 (14.17) | 13 (10.83) | 16 (13.33) | 7 (5.83) |
| Limited susceptibility | 1 (0.83) | 0 (0) | 2 (1.67) | 2 (1.67) |
| Definitive susceptibility | 2 (1.67) | 1 (0.83) | 0 (0) | 1 (0.83) |
| Marked susceptibility | 10 (8.33) | 15 (12.5) | 23 (19.17) | 10 (8.33) |

Abbreviations: *S. mutans*, *Streptococcus mutans*.

**Table S2.** The frequency of lactobacilli and *S. mutans* counts in dmft Groups (N=120).

|  | Minimum | Maximum | Mean | SD |
| --- | --- | --- | --- | --- |
| **dmft Group 1** (n=30) | | | | |
| **Lactobacilli count** (10^5^ CFU/ml) | 2 | 98 | 41.83 | 29.12 |
| ***S. mutans* count** (10^5^ CFU/ml) | 1 | 64 | 24.13 | 20.82 |
| **dmft Group 2** (n=29) | | | | |
| **Lactobacilli count** (10^5^ CFU/ml) | 16 | 250 | 82.41 | 51.06 |
| ***S. mutans* count** (10^5^ CFU/ml) | 5 | 162 | 55.28 | 39.41 |
| **dmft Group 3** (n=41) | | | | |
| **Lactobacilli count** (10^5^ CFU/ml) | 12 | 213 | 84.46 | 45.92 |
| ***S. mutans* count** (10^5^ CFU/ml) | 1 | 268 | 66.37 | 48.43 |
| **dmft Group 4** (n=20) | | | | |
| **Lactobacilli count** (10^5^ CFU/ml) | 30 | 280 | 87.25 | 50.70 |
| ***S. mutans* count** (10^5^ CFU/ml) | 12 | 240 | 62.70 | 51.10 |

Abbreviations: *S. mutans*, *Streptococcus mutans*; CFU, colony forming unit.

**Table S3.** The frequency of lactobacilli and *S. mutans* counts in the results of Snyder’s test (N=120).

|  | Minimum | Maximum | Mean | SD |
| --- | --- | --- | --- | --- |
| **Negative susceptibility** (n=53) | | | | |
| **Lactobacilli count** (10^5^ CFU/ml) | 2 | 280 | 55.08 | 48.82 |
| ***S. mutans* count** (10^5^ CFU/ml) | 1 | 268 | 43.66 | 55.58 |
| **Limited susceptibility** (n=5) | | | | |
| **Lactobacilli count** (10^5^ CFU/ml) | 33 | 87 | 82.41 | 22.99 |
| ***S. mutans* count** (10^5^ CFU/ml) | 12 | 88 | 55.28 | 31.73 |
| **Definitive susceptibility** (n=4) | | | | |
| **Lactobacilli count** (10^5^ CFU/ml) | 50 | 213 | 73.00 | 9.98 |
| ***S. mutans* count** (10^5^ CFU/ml) | 15 | 268 | 46.80 | 16.82 |
| **Marked susceptibility** (n=58) | | | | |
| **Lactobacilli count** (10^5^ CFU/ml) | 50 | 72 | 64.75 | 43.59 |
| ***S. mutans* count** (10^5^ CFU/ml) | 15 | 56 | 34.25 | 31.63 |

Abbreviations: *S. mutans*, *Streptococcus mutans*; CFU, colony forming unit.

**Table S4.** The frequency of lactobacilli and *S. mutans* PCRs in the results of Snyder’s test.

|  | **Snyder's test** (N=120) | | | |
| --- | --- | --- | --- | --- |
|  | **Negative susceptibility** (n=53) | **Limited susceptibility** (n=5) | **Definitive susceptibility** (n=4) | **Marked susceptibility** (n=58) |
|  | Count (%) | Count (%) | Count (%) | Count (%) |
| **Lactobacilli PCR** | | | | |
| Negative | 12 (10) | 0 (0) | 0 (0) | 4 (3.33) |
| Positive | 41 (34.17) | 5 (4.17) | 4 (3.33) | 54 (45) |
| ***S. mutans* PCR** | | | | |
| Negative | 42 (35) | 2 (1.67) | 4 (3.33) | 13 (10.83) |
| Positive | 11 (9.17) | 3 (2.5) | 0 (0) | 45 (37.5) |

Abbreviations: *S. mutans*, *Streptococcus mutans*.

**Table S5.** The frequencies of children’s daily habits and parents’ educational level in the PCRs and the counts of lactobacilli and *S. mutans*.

|  | | **Lactobacilli PCR** (N=120) | | ***S. mutans* PCR** (N=120) | | **Lactobacilli count** (N=120) | ***S. mutans* count** (N=120) |
| --- | --- | --- | --- | --- | --- | --- | --- |
|  |  | **Negative** | **Positive** | **Negative** | **Positive** | Mean (10^5^ CFU/ml) | Mean (10^5^ CFU/ml) |
|  |  | Count (%) | Count (%) | Count (%) | Count (%) |  |  |
| **Brushing teeth** (times) | 0 | 6 (5) | 35 (29.17) | 22 (18.33) | 19 (15.83) | 81.66 | 52.44 |
|  | 1 | 6 (5) | 60 (50) | 32 (26.67) | 34 (28.33) | 72.62 | 50.36 |
|  | 2 | 4 (3.33) | 8 (6.67) | 7 (5.83) | 5 (4.17) | 52.75 | 62.83 |
|  | 3 | 0 (0) | 1 (0.83) | 0 (0) | 1 (0.83) | 79 | 74 |
| **Sweet snacks** (times) | 1 | 7 (5.83) | 33 (27.5) | 19 (15.83) | 21 (17.5) | 66.6 | 57.43 |
|  | 2 | 7 (5.83) | 33 (27.5) | 27 (22.5) | 13 (10.83) | 68.27 | 42.95 |
|  | 3 | 0 (0) | 26 (21.67) | 13 (10.83) | 13 (10.83) | 81.35 | 50.23 |
|  | > 3 | 2 (1.67) | 12 (10) | 2 (1.67) | 12 (10) | 95.93 | 70.07 |
| **Main meals** (times) | 2 | 2 (1.67) | 14 (11.67) | 10 (8.33) | 6 (5) | 75.31 | 48.13 |
|  | 3 | 14 (11.67) | 74 (61.67) | 45 (37.5) | 43 (35.83) | 71.73 | 49.43 |
|  | > 3 | 0 (0) | 16 (13.33) | 6 (5) | 10 (8.33) | 83.5 | 73.88 |
| **Mothers’ education level** | Low education | 0 (0) | 12 (10) | 5 (4.17) | 7 (5.83) | 90.58 | 60.58 |
|  | High school | 2 (1.67) | 46 (38.33) | 23 (19.17) | 25 (20.83) | 79.88 | 61.77 |
|  | Undergraduate | 10 (8.33) | 29 (24.17) | 22 (18.33) | 17 (14.17) | 68.21 | 43.00 |
|  | Graduate | 4 (3.33) | 17 (14.17) | 11 (9.17) | 10 (8.33) | 60.57 | 44.43 |
| **Fathers’ education level** | Low education | 1 (0.83) | 16 (13.33) | 10 (8.33) | 7 (5.83) | 95.24 | 69.41 |
|  | High school | 3 (2.5) | 49 (40.83) | 20 (16.67) | 32 (26.67) | 84.5 | 62.4 |
|  | Undergraduate | 5 (4.17) | 22 (18.33) | 17 (14.17) | 10 (8.33) | 53.44 | 32.04 |
|  | Graduate | 7 (5.83) | 17 (14.17) | 14 (11.67) | 10 (8.33) | 58.21 | 42.17 |

Abbreviations: *S. mutans*, *Streptococcus mutans*; CFU, colony forming unit.

**Table S6.** The frequencies of children’s daily habits and parents’ educational level in the results of Snyder’s test.

|  | | **Snyder’s test** (N=120) | | | |
| --- | --- | --- | --- | --- | --- |
|  |  | **Negative susceptibility** (n=53) | **Limited susceptibility** (n=5) | **Definitive susceptibility** (n=4) | **Marked susceptibility** (n=58) |
|  |  | Count (%) | Count (%) | Count (%) | Count (%) |
| **Brushing teeth** (times) | 0 | 14 (11.67) | 3 (2.5) | 1 (0.83) | 23 (19.17) |
|  | 1 | 31 (25.83) | 1 (0.83) | 2 (1.67) | 32 (26.67) |
|  | 2 | 8 (6.67) | 1 (0.83) | 1 (0.83) | 2 (1.67) |
|  | 3 | 0 (0) | 0 (0) | 0 (0) | 1 (0.83) |
| **Sweet snacks** (times) | 1 | 15 (12.5) | 0 (0) | 0 (0) | 25 (20.83) |
|  | 2 | 22 (18.33) | 1 (0.83) | 2 (1.67) | 15 (12.5) |
|  | 3 | 12 (10) | 3 (2.5) | 2 (1.67) | 9 (7.5) |
|  | > 3 | 4 (3.33) | 1 (0.83) | 0 (0) | 9 (7.5) |
| **Main meals** (times) | 2 | 8 (6.67) | 0 (0) | 1 (0.83) | 7 (5.83) |
|  | 3 | 38 (31.67) | 4 (3.33) | 3 (2.5) | 43 (35.83) |
|  | > 3 | 7 (5.83) | 1 (0.83) | 0 (0) | 8 (6.67) |
| **Mothers’ education level** | Low education | 4 (3.33) | 1 (0.83) | 1 (0.83) | 6 (5) |
|  | High school | 19 (18.83) | 3 (2.5) | 2 (1.67) | 24 (20) |
|  | Undergraduate | 18 (15) | 0 (0) | 0 (0) | 21 (17.5) |
|  | Graduate | 12 (10) | 1 (0.83) | 1 (0.83) | 7 (5.83) |
| **Fathers’ education level** | Low education | 8 (6.67) | 0 (0) | 1 (0.83) | 8 (6.67) |
|  | High school | 18 (15) | 3 (2.5) | 2 (1.67) | 29 (24.17) |
|  | Undergraduate | 13 (10.83) | 1 (0.83) | 0 (0) | 13 (10.83) |
|  | Graduate | 14 (11.67) | 1 (0.83) | 1 (0.83) | 8 (6.67) |

**Table S7.** Results of Scheffe’s post hoc test for multiple comparisons of dmft groups on lactobacilli and *S. mutans* counts in children.

| **Lactobacilli count** (N=120) | | | | | | |
| --- | --- | --- | --- | --- | --- | --- |
| (I) dmft Group | (J) dmft Group | Mean Difference (I-J) | SE | *p* value | 95% CI (Lower Bound) | 95% CI (Upper Bound) |
| Group 1 | Group 2 | -40.58 | 11.615 | **0.009**^*^ | -73.53 | -7.63 |
|  | Group 3 | -42.63 | 10.716 | **0.002**^*^ | -73.03 | -12.23 |
|  | Group 4 | -45.417 | 12.875 | **0.008**^*^ | -81.94 | -8.89 |
| Group 2 | Group 1 | 40.58 | 11.615 | **0.009**^*^ | 7.63 | 73.53 |
|  | Group 3 | -2.05 | 10.822 | 0.998 | -32.75 | 28.65 |
|  | Group 4 | -4.836 | 12.964 | 0.987 | -41.61 | 31.94 |
| Group 3 | Group 1 | 42.63 | 10.716 | **0.002**^*^ | 12.23 | 73.03 |
|  | Group 2 | -2.05 | 10.822 | 0.998 | -28.65 | 32.75 |
|  | Group 4 | -2.787 | 12.165 | 0.997 | -37.3 | 31.72 |
| Group 4 | Group 1 | 40.58 | 11.615 | **0.009**^*^ | 8.89 | 81.94 |
|  | Group 2 | 4.836 | 12.964 | 0.987 | -31.94 | 41.61 |
|  | Group 3 | 2.787 | 12.165 | 0.997 | -31.72 | 37.3 |
| ***S. mutans* count** (N=120) | | | | | | |
| (I) dmft Group | (J) dmft Group | Mean Difference (I-J) | SE | *p* value | 95% CI (Lower Bound) | 95% CI (Upper Bound) |
| Group 1 | Group 2 | -31.143 | 10.799 | **0.045**^*^ | -61.78 | -0.51 |
|  | Group 3 | -42.233 | 9.963 | **0.001**^*^ | -70.5 | -13.97 |
|  | Group 4 | -38.567 | 11.971 | **0.019**^*^ | -72.53 | -4.61 |
| Group 2 | Group 1 | 31.143 | 10.799 | **0.045**^*^ | 0.51 | 61.78 |
|  | Group 3 | -11.09 | 10.062 | 0.75 | -39.64 | 17.46 |
|  | Group 4 | -7.424 | 12.053 | 0.944 | -41.62 | 26.77 |
| Group 3 | Group 1 | 42.233 | 9.963 | **0.001**^*^ | 13.97 | 70.5 |
|  | Group 2 | 11.09 | 10.062 | 0.75 | -17.46 | 39.64 |
|  | Group 4 | 3.666 | 11.31 | 0.991 | -28.42 | 35.75 |
| Group 4 | Group 1 | 38.567 | 11.971 | **0.019**^*^ | 4.61 | 72.53 |
|  | Group 2 | 7.424 | 12.053 | 0.944 | -26.77 | 41.62 |
|  | Group 3 | -3.666 | 11.31 | 0.991 | -35.75 | 28.42 |

Abbreviations: ^*^, Statistically significant; SE, standard error; dmft, decayed missing and filled (primary) teeth; *S. mutans*, *Streptococcus mutans*.

**Table S8.** Assessment of any possible correlation among age, lactobacilli count, and *S. mutans* count in dmft Groups using the Spearman’s Correlation Coefficient test.

|  |  |  | **Lactobacilli count** | ***S. mutans* count** | **Age** |
| --- | --- | --- | --- | --- | --- |
| **dmft Group 1**  (n=30) | Lactobacilli count | r | - | 0.848 | 0.363 |
|  |  | 95% CI (lower; upper) | - | 0.697; 0.927 | -0.008; 0.646 |
|  |  | *p* value | - | **< 0.001**^*^ | **0.048**^*^ |
|  | *S. mutans* count | r | 0.848 | - | 0.38 |
|  |  | 95% CI (lower; upper) | 0.697; 0.927 | - | 0.012; 0.658 |
|  |  | *p* value | **< 0.001**^*^ | - | **0.038**^*^ |
|  | Age | r | 0.363 | 0.38 | - |
|  |  | 95% CI (lower; upper) | -0.008; 0.646 | 0.012; 0.658 | - |
|  |  | *p* value | **0.048**^*^ | **0.038**^*^ | - |
| **dmft Groups 2-4**  (n=90) | Lactobacilli count | r | - | 0.514 | 0.313 |
|  |  | 95% CI ((lower; upper) | - | 0.338; 0.656 | 0.107; 0.493 |
|  |  | *p* value | - | **< 0.001**^*^ | **0.003**^*^ |
|  | *S. mutans* count | r | 0.514 | - | 0.258 |
|  |  | 95% CI (lower; upper) | 0.338; 0.656 | - | 0.048; 0.447 |
|  |  | *p* value | **< 0.001**^*^ | - | **0.014**^*^ |
|  | Age | r | 0.313 | 0.258 | - |
|  |  | 95% CI (lower; upper) | 0.107; 0.493 | 0.048; 0.447 | - |
|  |  | *p* value | **0.003**^*^ | **0.014**^*^ | - |
| **All dmft Groups**  (N=120) | Lactobacilli count | r | - | 0.662 | 0.389 |
|  |  | 95% CI (lower; upper) | - | 0.544; 0.755 | 0.221; 0.535 |
|  |  | *p* value | - | **< 0.001**^*^ | **< 0.001**^*^ |
|  | *S. mutans* count | r | 0.662 | - | 0.352 |
|  |  | 95% CI (lower; upper) | 0.544; 0.755 | - | 0.179; 0.504 |
|  |  | *p* value | **< 0.001**^*^ | - | **< 0.001**^*^ |
|  | Age | r | 0.389 | 0.352 | - |
|  |  | 95% CI (lower; upper) | 0.221; 0.535 | 0.179; 0.504 | - |
|  |  | *p* value | **< 0.001**^*^ | **< 0.001**^*^ | - |

Abbreviations: ^*^, Statistically significant; r, Spearman’s rho; CI, Confidence interval; dmft, decayed missing and filled (primary) teeth; *S. mutans*, *Streptococcus mutans*.

**Table S9.** Estimation of Odds Ratio for bacterial PCR in dmft Groups.

|  | **Comparison** | **OR** | **95%CI (lower; upper)** | **Z** | ***p* value** |
| --- | --- | --- | --- | --- | --- |
| **Lactobacilli PCR** (N=120) | dmft Groups 1 and 2 | 7.816 | 1.551; 39.373 | 2.492 | **0.013**^*^ |
|  | dmft Groups 1 and 3 | 7.333 | 1.826; 29.452 | 2.809 | **0.005**^*^ |
|  | dmft Groups 1 and 4 | 11 | 1.289; 93.835 | 2.192 | **0.028**^*^ |
|  | dmft Groups 1 and 2-4 | 8.105 | 2.665; 24.653 | 3.687 | **0.002**^*^ |
| ***S. mutans* PCR** (N=120) | dmft Groups 1 and 2 | 1.223 | 0.495; 3.981 | 0.637 | 0.524 |
|  | dmft Groups 1 and 3 | 2.699 | 1.021; 7.135 | 2.002 | **0.045**^*^ |
|  | dmft Groups 1 and 4 | 1.727 | 0.548; 5.449 | 0.932 | 0.351 |
|  | dmft Groups 1 and 2-4 | 1.974 | 0.843; 4.62 | 1.568 | 0.117 |

Abbreviations: ^*^, Statistically significant; OR, Odds ratio; dmft, decayed missing and filled (primary) teeth; *S. mutans*, *Streptococcus mutans*.
